# Primary care providers as a critical access point to HIV information and services for African American and Latinx communities

**DOI:** 10.1371/journal.pone.0246016

**Published:** 2021-02-04

**Authors:** Gregory Carter, Brennan Woodward, Anita Ohmit, Andrew Gleissner, Meredith Short

**Affiliations:** 1 Indiana University School of Nursing Bloomington, Bloomington, IN, United States of America; 2 Indiana Minority Health Coalition, Indianapolis, IN, United States of America; 3 Positive Link Bloomington Indiana, Bloomington, IN, United States of America; Hunter College, UNITED STATES

## Abstract

**Purpose:**

This study aimed to examine the association between confidence in accessing HIV services, primary sources of HIV information, and primary care provider status for African American and Latinx individuals in Indiana.

**Methods:**

An online survey was disseminated to African American and Latinx individuals using snowball and social media recruitment methods, resulting in a final sample size of n = 308. A multivariable linear regression analysis was performed to examine the relationships between confidence accessing HIV services, primary care provider status, sexual identification, and sources of HIV information.

**Results:**

Of the total respondents, 62.5% (n = 193) identified as male and 36.9% (n = 114) identified as female. Most identified as African American (72.5%, n = 224), followed by 27.2% (n = 84) who identified as Latinx. Participants who used their primary care providers as a primary source of obtaining HIV information had a significantly higher level of comfort with accessing HIV services. Those who identified family members as a primary source of HIV information and those who identified as bisexual demonstrated a lower level of confidence in accessing HIV services.

**Discussion:**

This study's results enhance our understanding of marginalization within minority groups regarding sexual identification and accessing HIV services. These results also offer insight into the importance of healthcare access because having a primary care provider was a strong predictor of increased confidence in accessing HIV services.

## Introduction

Nearly four decades after the first cases of AIDS were reported, HIV continues to be a public health concern, and one of the most significant issues facing minorities in the United States. Between 2010 and 2017, the number of new HIV diagnoses among adults and adolescents in the United States decreased by 11% [1]. These findings suggest that HIV prevention and treatment efforts such as pre-exposure prophylaxis (PrEP), community-based education efforts, and universal HIV testing are making a significant impact. However, despite recent advances in HIV reduction efforts, the effect has not been equitable across populations.

While the overall rate of HIV diagnosis among African Americans has declined recently, African Americans continue to account for 42% of new cases of HIV. More specifically, African American men who have sex with other men (MSM) ages 25–34 are the population most affected by HIV, and in this population, new diagnoses have increased 42% between 2010–2017 [2]. Linking HIV-positive individuals with treatment during the early stages of infection is critical to maintaining health and quality of life [3]; yet, 1 in 7 African Americans are unaware they are living with HIV [4], meaning they cannot receive current standards of treatment, may unknowingly pass HIV on to others, and are at risk of premature death associated with AIDS-related illnesses [5].

The prevalence of individuals living with HIV continues to be lower among Latinx individuals than among African Americans; however, adult Latinx individuals account for 26% of new HIV diagnoses [6], with cases among Latinx MSM increasing 21% Between 2010–2017. [6] Similar to African Americans, 1 of every 6 HIV-positive Latinx individuals remain unaware of their HIV-positive serostatus, ultimately precluding them from accessing treatment services, placing them at increased risk for premature death, and inadvertently increasing the opportunity for passing on the virus to sexual partners [6].

The Centers for Disease Control and Prevention (CDC) HIV-testing guidelines recommend that each person between the ages of 13 and 64 be tested for HIV at least once, with individuals in higher-risk groups receiving more frequent testing [7]. Despite the guidelines, the CDC reports approximately 162,000 Americans are unaware of their HIV-positive serostatus. This lack of awareness regarding HIV status is a significant concern because approximately 40% of new HIV diagnoses are transmitted by an individual unaware of being HIV-positive [7]. Engagement with a primary care provider is essential to obtaining HIV testing and improving health outcomes associated with HIV treatment and prevention [8, 9].

Anxiety and decreased self-efficacy regarding accessing HIV services continue to be primary barriers to obtaining timely HIV testing [10, 11]. For HIV-negative individuals, routine testing extends beyond the awareness of HIV status; it offers the opportunity to receive prevention education and initiation of prevention regimens such as PrEP. This has significant public health implications as previous studies have demonstrated that adherence to a PrEP regimen can prevent the acquisition of HIV by greater than 90% among those at significant risk [12, 13]. However, despite the positive implications of HIV testing and prevention efforts, the extant literature cannot adequately describe the association between sources of HIV information and self-efficacy in accessing HIV services.

Equitable access to healthcare continues to be a significant concern in Indiana, where people of color disproportionately experience suboptimal health-associated outcomes. Black and Latinx individuals in Indiana are more likely to report being uninsured, with 13% of Black and 21% of Latinx adults lacking health insurance compared with 8% of their white counterparts [14]. Furthermore, approximately 40% of Latinx and 25.4% of Black adults in Indiana report not having access to a primary care provider, compared with only 17.8% of white individuals. In Indiana, people of color are also more likely to report not seeing a healthcare provider in the previous year, with 15.2% of Black and 19.2% of Latinx adults reporting not seeing a healthcare provider in the previous 12 months, impeding access to HIV standards of care [15].

Black and Latinx individuals represent approximately 9% and 7% of the Indiana population, respectively; however, they account for 46% of those living with HIV [16]. Black adults in Indiana represent 825.1 per 100,000, and Latinx adults represent 312.1 per 100,000 of those living with an HIV diagnosis, compared with 120.7 per 100,000 of white adults. Black adults are also more likely to die from HIV and AIDS-associated illness in Indiana, where the HIV mortality rate is approximately five times greater for Black adults (11.6 per 100,000) compared to white adults (2.5 per 100,000) [16].

In light of the established relationship between proactive HIV testing and prevention measures and existing barriers to care, the present study examined the association between confidence accessing HIV services, primary sources of HIV information, and primary care provider status among African American and Latinx individuals in Indiana, with potential implications for targeted interventions to improve the uptake of HIV testing, treatment, and prevention outcomes.

## Methods

### Design and subjects

All study materials and procedures were approved by the Indiana University Institutional Review Board (IRB protocol No. 1911184919). The present study employed a community based participatory design to ensure the voices and experiences of the Black and Latinx communities drove the research [17]. Initially, a community advisory board was assembled to identify salient issues and research questions about HIV prevention and treatment among Black and Latinx adults in Indiana. The advisory board included ten individuals, six who identified as Black and four who identified as Latinx. The members represented the northern, central, and southern regions of the state. The primary investigator facilitated the discussion by allowing each member to identify a component of HIV care access. The discussion resulted in the identification of three overarching themes: barriers to primary care, uncertainty accessing HIV services, and where people of color access information regarding HIV prevention and treatment. The themes guided the development of the online survey. After researchers developed the survey, it was taken back to the advisory board for review. During the final review process, board members offered comments and feedback, which resulted in the final 37 item survey.

A cross-sectional study was fielded from February 27 through March 14, 2020. Inclusion criteria were as follows: 1) at least 18 years of age, 2) identify as African American or Latinx, and 3) a resident of Indiana. We recruited participants by using a convenience sampling approach via a digital flyer posted on social media sites and through physical recruitment flyers placed in healthcare settings, community centers, libraries, and ethnic markets throughout the state. A snowball sampling approach was implemented where each participant who completed the survey would receive a brief thank you email that included 1) a $10.00 electronic gift card in exchange for their time, and 2) a request to forward the survey link to acquaintances or family members who may qualify for the study. Materials were available in English and Spanish to increase representation among non-native English-speaking participants. A professional translation service was used for the translation of study materials.

Before they could begin the survey, each participant was asked to read the study information sheet and select “agree to participate” before initiating the survey. To identify participants with a low attention to instructions and detail, we inserted an instructional manipulation question in the middle of the survey; for this question, participants were instructed to answer with the response “Yellow” [18, 19]. Before the close of the study, 358 individuals had accessed the survey. Of those who initiated the survey, 5 (1.4%) did not meet the inclusion criteria. Of those participants who did not meet the inclusion criteria, 3 (0.8%) identified as white and 2 (0.5%) lived outside Indiana. An additional 30 participants (8.5%) did not progress past the demographic questions, and 6 participants (1.7%) did not pass the instructional manipulation question. Of the remaining participants, 3 evidenced missing data values in sexual identification and 1 did not indicate primary care provider status and were subsequently removed from analysis, yielding a final sample size of 308. The majority of the participants chose to complete the survey in English (n = 300); however, 8 participants completed the survey in Spanish. Of those who chose the Spanish version, six identified as female, and two identified as male. Regarding age, 2 of the females who selected to take the survey in Spanish were between the ages of 18–24, 3 were between 25–34, and 1 was between the ages of 55–64. Both males identified their ages between 25–34.

### Measurements

The survey asked questions about participant sociodemographics including age, gender, level of education, HIV testing history, provider-recommended HIV screening, and county of residence. The outcome variable, level of confidence accessing HIV services, was assessed by one question developed for this survey asking participants to identify their confidence accessing HIV services. Participants were rated their confidence on a scale ranging from 0 to 100, with 0 indicating the lowest level of confidence and 100 indicating the highest level of confidence. Access to a primary care provider was assessed with one question developed for this survey which participants were asked to choose one of three options: Yes (the participant has a primary care provider), No (the participant does not have a primary care provider), or Unsure (the participant is unsure if they have a primary care provider). To identify the primary sources from which participants obtained HIV information, the survey prompted participants to select from a list of 9 potential options. The sources of HIV information were identified with input from the community advisory board, community research partners, and members of the research team. Finally, sexual identity was assessed with one question developed for this survey, asking each participant to select one of three options: straight, gay, or bisexual.

### Statistical analysis

Data analysis was conducted in 2 phases. First, all data were analyzed descriptively by use of univariate analysis. Second, a multivariable linear regression was used to examine the scores reflecting confidence accessing HIV services as a function of the predictor variables included in the multivariable model. Cases were weighted by age group. Statistics Package for the Social Sciences version 26 (SPSS Inc) was used for all analyses. Before data analysis an examination of test assumptions indicated a satisfactory level of normality, linearity, and homoscedasticity.

## Results

Of the 308 participants, 62.5% (n = 193) identified as male and 36.9% (n = 115) identified as female. The majority were between the ages of 25 and 34 years (n = 143, 46.3%) and 23.9% (n = 74) were in the 18–24 age group. Most identified as African American (72.5%, n = 224), followed by 27.2% (n = 84) who identified as Latinx. Almost half stated they were heterosexual (47.9%, n = 148), 35.3% (n = 109) identified as gay, and 15.5% (n = 46) identified as bisexual. A complete description of participant demographics can be found in Table 1.

**Table 1:** Demographics of the African American and Latinx survey (n = 308), 2020.

	Ethnic Group			
	Latinx		African American	
Variable	N	%*	N	%*
**Gender**				
Male	38	12.3	155	50.3
Female	46	14.9	69	22.4
**Age, years**				
18–24	24	7.7	50	16.2
25–34	40	12.9	103	33.4
35–44	12	3.8	55	17.8
45–54	6	1.9	15	4.8
55–64	2	0.6	1	0.3
**Education**				
Less Than High School	2	0.6	6	1.9
High School	7	2.2	17	5.5
Some College	16	5.1	36	11.6
Associate Degree	18	5.8	60	19.4
Bachelor’s Degree	37	12	93	30.1
Master’s Degree	3	0.9	8	2.5
Doctoral Degree	1	0.3	4	1.2
**Sexual Identification**				
Straight	45	14.8	103	33.9
Gay	23	7.5	86	28.3
Bisexual	13	4.2	33	10.7
**Previous HIV Test**				
Yes	46	15.1	127	41.7
No	37	12.1	94	30.9
**Has a PCP Recommended You Have an HIV Test**				
Yes	30	9.7	141	45.7
No	54	17.5	83	27.5
**Source of HIV Information**				
Primary care provider	30	35.7	98	43.7
Public Health Clinic	34	41.6	125	55.8
Social Media	62	73.8	136	60.7
Friends	102	82.3	40	17.8
Family	25	29.7	59	26.3
School	21	25	34	15.1
Church	19	22.6	46	20.5
Online	39	46.4	95	42.4
Media (TV, books, etc.)	16	19	33	14.7

*represents the percent of the total n = 308.

Our multivariable model (Table 2) indicated that, with control for sexual identification and primary care provider status, level of confidence in accessing local HIV services was related to obtaining HIV information from a primary care provider at a statistically significant level with a medium effect size (η^2^ = .07). The results indicated that the regression model was statistically significant (p < .001) and explained 14% (R Square = .143, Adjusted R Square = .103) of the variance in the dependent variable of comfort with accessing local HIV services. Identifying as bisexual was the strongest predictor in the model examining confidence with accessing HIV services (β = -.287)

**Table 2:** Results of multivariable linear regression analysis examining confidence accessing local HIV services (n = 308).

Variable	*B*(*SE*)	β	95% CI	*p*
**Sexual Identification**				
Straight	-	-	-	-
Gay	-.705(2.76)	-.015	(-6.13, 4.72)	.79
Bisexual	-14.66(3.62)	-.237	(-21.78, -7.55)	< .001*
**Primary Care Provider Status**				
Yes	-	-	-	-
No	-7.16(2.99)	-.150	(-13.05, -1.27)	.017*
Not Sure	5.84(3.21)	.116	(-.48, 12.16)	.070
**Sources of Primary HIV Information**				
Primary Care Provider	5.87(2.65)	.131	(.657, 11.08)	.027*
Public Health Clinic	-1.33(2.49)	-.030	(-6.22, 3.57)	.594
Social Media	1.73(2.69)	.037	(-3.56, 7.02	.520
Friends	.574(2.56)	.013	(-4.47, 5.61)	.823
Family	-7.18(2.85)	-.144	(-12.79, -1.57)	.012*
School	-1.31(3.27)	-.032	(-7.74, 5.13)	.690
Church	.615(3.04)	.011	(-5.36, 6.59)	.840
Online	-1.59(2.67)	-.036	(-6.85, 3.66)	.550
Other Media (television, magazines, etc.)	4.31(3.59)	.072	(-2.76, 11.38)	.231

The results indicated that those participants who used their primary care providers as a primary source of HIV information had a significantly higher level of comfort with accessing HIV services (B = 5.86, SE = 2.65, β = .13, p = .027) compared with those who did not view their primary care provider as a main source of information. Participants who identified family members as a primary source of HIV information had a significantly lower level of confidence with accessing HIV services (B = -7.18, SE = 2.85, β = -.14, p = 0.27) than were those who did not use their family members as a primary source of HIV information. Identifying as bisexual was significantly associated with a lower level of confidence with accessing HIV services (B = -14.66, SE = 3.62, β = -.29, p = < .001), in reference to those who identified as straight. Finally, not having a primary care provider was significantly associated with a lower level of confidence in accessing local HIV services (B = -7.16, SE = 2.99, β = -.15, p = .017) in reference to those individuals who had a primary care provider.

Study results were shared with the community advisory board and key community partners representing the northern, central, and southern regions of the state. Results were also discussed individually with community partners during a one-on-one debrief via video conferencing. Key study outcomes were presented to help inform the discussion and potential implications for African American and Latinx communities.

## Discussion

This study set out to assess the relationship between a person’s primary source of HIV information, sexual identification, and having a primary care provider and the level of comfort with accessing HIV services among African American and Latinx men and women in Indiana. We hypothesized that individuals who viewed their primary care provider as a primary source for HIV information would also feel more comfortable accessing HIV services. The results confirm that respondents who had a primary care provider and viewed them as a primary source of HIV information were significantly more confident accessing HIV services than were those who did not view their primary care provider as primary source of HIV information. It is possible, therefore, that by aligning provider practices with CDC guidelines we can see a more equitable reduction in HIV.

Having access to a usual source of care is an important factor in improving HIV-related outcomes. This result echoes those of previous studies which reported that people without access to a primary care provider were more likely to experience adverse health outcomes [20, 21]. This is particularly relevant in Indiana where approximately 25% percent of African Americans and 39.2% percent of Hispanics lack access to a usual source of care [22]. However, it also possible that this result is a function of a complex collection of barriers, including a lack of health insurance. Previous research exploring the impact of insurance status on HIV care discovered that insurance coverage is a protective factor against HIV transmission [23]. The present finding also seems consistent with previous studies which reported that people of color who viewed their healthcare provider as a primary source of information were more motivated to adopt positive behaviors regarding HIV care [24].

Using a family member as a primary source of HIV information was significantly associated with a lower level of confidence in accessing HIV services. This finding appears to follow those of previous studies showing that among people of color, HIV stigma was a significant barrier to accessing HIV services and disclosing HIV-positive serostatus [25–27]. However, despite facing stigma and homophobia from family members, previous studies have reported that African Americans prefer that their sexual health education come from family members. The preference for family-based education was attributed to the belief that sources of information outside of family and friends are inaccurate [25]. The increased experience of stigma combined with a cultural distrust of healthcare providers may ultimately lead to avoidance of HIV services, inadvertently increasing the risk of acquiring or transferring HIV.

Higher degrees of HIV knowledge are often associated with an increased level of confidence in accessing HIV services and a history of receiving at least one HIV test [28, 29]. While the results of the present study cannot describe the level of HIV knowledge among the participants, a possible explanation for a decreased level of confidence in accessing HIV services among those who view family members as a source of HIV information may be a general lack of HIV knowledge. Future research exploring the content and context of the HIV information provided by family members is needed to better understand whether this results from medical misinformation being passed among family members or whether using family members as a primary source of HIV information is a sign of a larger distrust of the medical system. The latter could lead to a decreased level of comfort with accessing health services in general, and not just those related to HIV.

Stigma is of particular concern for people of color who identify as gay and bisexual. They report more medical mistrust and are more hesitant to accept HIV prevention services than white gay and bisexual men. One of the primary reasons Black gay and bisexual men are more reluctant to engage with HIV services is an "intense" experience of stigma against the LGBT population [30]. As previously discussed, our study found that identifying as bisexual was associated with a lower level of confidence in accessing HIV services. Therefore, altering the discussion about HIV may be one way to improve access to HIV prevention and treatment services. Specifically, shifting the conversation about HIV to be more inclusive of disparate populations, including those who identify as heterosexual, may alleviate some of the stigma associated with HIV and the LGBT community. Another implication is to address the lack of LGBT cultural competency among healthcare providers [30]. Improving healthcare provider's understanding and interactions with LGBT patients may eliminate some barriers reported by our Bisexual participants.

Regarding the impact of sexual identity in the context of confidence accessing HIV services, identifying as bisexual was significantly associated with a lower level of confidence accessing HIV services than were those who identified as straight. This is an important finding for bisexual minorities as the rate of new HIV infections continues to increase in this population [31]. And bisexual people of color experience more medical mistrust, which serves as an additional barrier to HIV testing [32]. Yet, what continues to remain unclear is the convergence of factors that contribute to African American and Latinx bisexuals experiencing a greater sense of discomfort accessing HIV services.

The present study has several limitations worth noting. First, the sample was restricted to African American and Latinx individuals who live in Indiana. As a result, the findings may not be generalizable to all Black and Latinx individuals within or outside of Indiana. Second, the findings were limited by the use of a cross-sectional design. Specifically, the accuracy of the responses was subject to response bias. Third, the study employed a cross-sectional design, and as such causality cannot be determined. Fourth, the study did not measure insurance status and as a result, we are not able to describe the relationship between health insurance status and access to healthcare. Finally, although the study has successfully demonstrated multiple barriers and facilitators to accessing HIV services, it has certain limitations in terms of an extensive examination of variables that contribute to healthcare access, including measures from other domains such as HIV knowledge, and previous healthcare experiences.

Overall, the results suggest that the primary source of HIV information for African American and Latinx individuals matters in the context of attitude regarding accessing HIV information. Having a primary care provider and using that provider as a source of HIV information are important factors for increasing an individual’s level of confidence in accessing HIV services. The comparison of straight, gay, and bisexual identification showed that, compared with straight men and women, identifying as bisexual was the strongest predictor and was associated with a significantly lower degree of confidence in finding and accessing HIV services.

The results of this study enhance our understanding of marginalization within minority groups about sexual identification and accessing HIV services. Results from this study also offer insight into the importance of healthcare access. The relevance of having a primary care provider for confidence in accessing HIV services suggests the importance of a closer inspection of people of color without access to such a provider.
